# Comments on “Reflections on the current context and evaluation of undergraduate medical education”

**DOI:** 10.1590/0100-6991e-20243858-en

**Published:** 2024-12-02

**Authors:** VANIA FONTANELLA, MARIA CELESTE MORITA

**Affiliations:** 1 - Universidade Federal do Rio Grande do Sul, Associação Brasileira de Ensino Odontológico - Porto Alegre - RS - Brasil; 2 - Universidade Estadual de Londrina, Associação Brasileira de Ensino Odontológico - Londrina - PR - Brasil

**Keywords:** Health Human Resource Training, General Practitioners, Licensure, Capacitação de Recursos Humanos em Saúde, Generalistas, Licenciamento

## Abstract

The authors debate the arguments presented in the editorial “Reflections on the current context and evaluation of undergraduate medical education”, bringing the context that culminated in the recent application of the National Proficiency Exam in Dentistry.

## RESUMO

As autoras debatem os argumentos expostos no editorial “Reflexões acerca do contexto atual e da avaliação da formação médica na graduação”, trazendo o contexto que culminou na recente aplicação do Exame Nacional de Proficiência em Odontologia.

In an article published in the Journal of the Brazilian College of Surgeons^1^, the authors discuss the expansion of general practitioner training in Brazil and the possible reasons why this phenomenon has not found a parallel in the search for admission to Medical Residency Programs. Additionally, it addresses the terminality characteristic of medical training in undergraduate courses, the systems for evaluating the quality of training used internationally for licensing, and the recent Bill 2.294/20242, which proposes the National Exam of Proficiency in Medicine. At the invitation of the editor, we discuss what is exposed in the article, bringing the context that culminated in the recent application of the National Dental Proficiency Exam.

Conducting a Proficiency Exam in the health area has always been a controversial topic. A simple search on the web results in a series of contrary manifestations from different and relevant actors. Even in the cases of other areas, in which the exam is supported by specific legislation, such as the exam of the Brazilian Bar Association (OAB) and the sufficiency exam of the Federal Accounting Council (CFC), this procedure has to be understood as the result of a process, in which one must weigh the interests of students, their families, and society. In the two examples cited, a historical retrospective is necessary to understand that an examination of this nature, with the purpose of establishing an additional stage to the diploma registration, gives rise to debate and begets defenders and critics. 

The OAB exam, created in 1963 by Law No. 4,2153, provided that for registration as a lawyer in the Federal Council of the OAB, the applicant could present a certificate proving the attendance in an internship or approval in the Bar Exam. That law was later replaced by Law No. 8,906/19944, no longer accepting the internship as an access to OAB membership. Thus, only from then on, the university diploma of the undergraduate course per se did not qualify the Bachelor of Law to practice law. 

In the case of the CFC, the first edition of the sufficiency exam was held in 2000, based on the CFC Resolution No. 853/1999, but lasted only until 2004, as the lack of legal support for the exam precipitated lawsuits that challenged the competence of the Federal Accounting Council to establish such a measure. It was only after the federal law 12.249 of 2010 that the exam effectively became mandatory. 

Both in the case of the Federal Council of the OAB and the CFC, the motivation for the examination was protecting society from professionals without due preparation for their practice. Whether this objective is achieved and to what extent is another discussion that also mobilizes those for and against. 

In health, especially Medicine, is it not necessary to protect society from unprepared professionals too? Probably, this answer would be unanimously yes, although the strategy established by different Professional Councils to answer it may bring to the fore new and old discussions. 

Recently, in 2022, the first initiative of a proficiency exam in Dentistry had an experimental phase through a pilot project with undergraduates in the State of Paraná. Most likely, the context that fostered the implementation of this measure is very similar to what occurs in other health professions: an exponential increase in undergraduate courses, disorderly expansion leading to the worsening of regional asymmetries in the distribution of professionals, and an evaluation system that is insufficient to guarantee training quality. 

A study on what has happened from 1859, the date of the first undergraduate course in Dentistry, to 20205 showed that the expansion of supply introduced many problems, such as the waste of resources, as idle vacancies and the accumulated dropout rate, inexpressive in the past, began to expand. The study also pointed out that the National System for the Evaluation of Higher Education, designed to be the guarantor of the desirable training quality and the regulation of the supply expansion, has many limitations. Among these, the divergences regarding the adequacy of student training to the National Curriculum Guidelines^6^ and the authorization processes for new courses stand out. For the authorization of new courses, the study showed that when considering the parameters adopted by the reviewers of the National Health Council, which are based on social need as a founding element, only one third of the courses authorized by the Ministry of Education would have been effectively licensed. 

In the case of the undergraduate’s evaluation by the National Student Performance Evaluation Exam (Enade), we agree with the authors of the article in question. There are important biases in the results, influenced by several factors, such as the selection by convenience of the best students to take the test or the way the concept is generated by intragroup comparison, in addition to many other problems already pointed out by the media.

In the case of the undergraduate’s evaluation by the National Student Performance Evaluation Exam (Enade), we agree with the authors of the article in question. There are important biases in the results, influenced by several factors, such as the selection by convenience of the best students to take the test or the way the concept is generated by intragroup comparison, in addition to many other problems already pointed out by the media.

In view of this situation, the introduction of a measure to assess the knowledge necessary for profes- sional practice presents itself as a valuable tool to add transparency to the level of preparation of those who enter the labor market. The adoption of a Proficiency Exam outside the walls of educational institutions, pre- pared in close tandem with the technical and ethical as- pects related to the performance of the generalist, will certainly not be the panacea for all issues, but it will pro- vide society with a diagnosis that, as part of a process, will contribute to the quality of training in undergradu- ate courses.
